# A Lab-Scale Evaluation of Parameters Influencing the Mechanical Activation of Kaolin Using the Design of Experiments

**DOI:** 10.3390/ma17184651

**Published:** 2024-09-23

**Authors:** Jofre Mañosa, Adrian Alvarez-Coscojuela, Alex Maldonado-Alameda, Josep Maria Chimenos

**Affiliations:** 1 DIOPMA Research Group, Department of Materials Science and Physical Chemistry, Universitat de Barcelona, Martí i Franquès 1, 08028 Barcelona, Spain; aalvarez22@ub.edu; 2 Fundación Centro Tecnológico de Investigación Multisectorial (CETIM), Parque Empresarial de Alvedro, 15180 Culleredo, Spain; amaldonado@cetim.es

**Keywords:** clay mechanical activation, kaolin, kaolinite, design of experiments, milling parameters, supplementary cementitious materials, amorphization

## Abstract

This research investigates the mechanical activation of kaolin as a supplementary cementitious material at the laboratory scale, aiming to optimize milling parameters using the response surface methodology. The study evaluated the effects of rotation speed and milling time on the amorphous phase content, the reduction in crystalline kaolinite, and impurity incorporation into the activated clay through the Rietveld method. The results demonstrated that adjusting milling parameters effectively enhanced clay activation, which is crucial for its use in low-carbon cements. High rotation speeds (300/350 rpm) and prolonged grinding times (90/120 min) in a planetary ball mill increased the pozzolanic activity by boosting the formation of amorphous phases from kaolinite and illite and reducing the particle size. However, the results evidenced that intermediate milling parameters are sufficient for reaching substantial degrees of amorphization and pozzolanic activity, avoiding the need for intensive grinding. Exceedingly aggressive milling introduced impurities like ZrO_2_ from the milling equipment wear, underscoring the need for a balanced approach to optimizing reactivity while minimizing impurities, energy consumption, and equipment wear. Achieving this balance is essential for efficient mechanical activation, ensuring the prepared clay’s suitability as supplementary cementitious materials without excessive costs or compromised equipment integrity.

## 1. Introduction

The cement industry is a major contributor to pollution in the construction sector due to the carbon- and energy-intensive production of clinker, the key component of Portland cement (PC). Clinker manufacturing accounts for 5–8% of global CO_2_ emissions and 7–15% of global industrial energy demand, making it a significant source of greenhouse gas emissions [1,2]. Most energy consumption stems from fossil fuel combustion [3,4], while clinker calcination, driven by limestone decomposition and fuel burning, is the main source of CO_2_ emissions [5,6]. Efforts to reduce the environmental impact of PC production have led to extensive research on eco-friendly alternatives.

In the short term, strategies to reduce the cement industry’s environmental impact prioritize improving clinker production processes or reducing clinker usage. Enhancements in clinker production can be achieved through process efficiency improvements, fuel and feedstock switching, and carbon capture and storage [7,8,9,10], while options like using alternative cementitious materials (ACMs) [11] or partially substituting clinker offer viable approaches to reducing clinker usage [12,13].

Accordingly, replacing clinker or PC with supplementary cementitious materials (SCMs) has emerged as a promising short- and medium-term solution for decarbonizing the cement industry [14]. SCMs, which include materials such as fly ash, slags, natural pozzolans, silica fume, and limestone, are valued for their high reactivity and ability to improve cement properties while reducing costs, decreasing carbon footprint, and enhancing durability [15,16]. However, the availability of secondary sources such as fly ash and slags is declining, making natural pozzolans an attractive alternative due to their abundant supply. In this sense, clay minerals are particularly promising as potential SCMs because they can quickly meet the demands of the cement industry [17].

The chemical effect of SCMs involves the silica and alumina compounds present in the SCM, which react with calcium hydroxide and water to form mainly calcium (alumino)silicate hydrate (C(A)SH), the main hydration product of cement. While C(A)SH is the predominant reaction product, other calcium aluminate hydrates or crystalline calcium aluminosilicate hydrates are commonly detected in blended cement containing clay minerals [18]. However, the strong crystalline structure of clay minerals limits their reactivity and hinders their direct use as SCMs. To address this constraint, clays are often modified through activation processes to enhance their reactivity, making them suitable as SCMs. The most common method for enhancing the reactivity of clay minerals is thermal activation, which involves calcining the clay. This process removes water and hydroxyl groups within clay minerals, inducing structural distortion, and some clays become amorphous due to the collapse of their crystalline structure [19,20].

**Figure 1:**
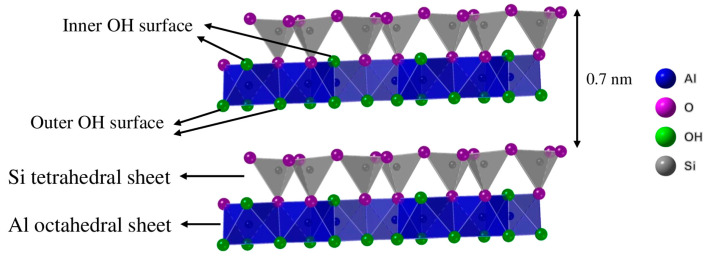
Kaolinite layered structure.

Most advances in using clays as SCMs have focused on kaolinitic clays due to their widespread availability and high reactivity values after thermal activation [21]. Kaolinite is characterized by a laminar structure with layers comprised of silicon tetrahedra and aluminum octahedra sheets (see Figure 1). The layers are linked via hydrogen bonds in the interlayer. Despite dehydroxylation occurring at around 400–650 °C, kaolinite (Kaol) thermal activation is typically performed at higher temperatures, usually between 700 and 800 °C, to enhance the amorphization of Kaol’s structure [22,23]. Given the lower temperature requirements compared to the clinkerization process, the thermal activation of kaolinitic clays can potentially reduce the carbon footprint and energy consumption of the cement industry by partially replacing clinker. However, with the current technology, the kiln temperatures required for industrial calcination can only be achieved by burning fossil fuels [24].

Mechanical activation emerges as a viable alternative to thermal activation for obtaining highly reactive clays, consuming only electricity and thus potentially reducing CO_2_ emissions, depending on the region’s electrical mix. Additionally, this method could reduce the total energy consumption of the activation process [25]. Mechanical activation induces the amorphization of the Kaol structure and reduces its particle size through the breakage of chemical and physical bonds. This structural distortion increases reactivity and pozzolanic activity, making the material suitable as an SCM [26,27,28,29].

The mechanical activation of kaolin is already a reality at the industrial level [30]. However, research gaps remain to be addressed, and many aspects and considerations still need to be explored at the laboratory scale to further investigate its properties as an SCM. This indicates that significant research work remains to be done at the lab scale, beginning with the proper activation of kaolin before the technology can be adopted globally at the industrial scale. This activation depends on various parameters that directly influence the pozzolanic activity and overall reactivity of mechanically treated clay, including milling time, milling speed, jar filling, ball size, and load amount [31].

In this research, an experimental design was conducted at the laboratory scale to investigate the impact of milling time and rotation speed in a planetary ball mill on the mechanical activation of clay. The main goal was to optimize the milling process and assess the influence of milling parameters on the properties of mechanically activated clay. Specifically, this study aimed to evaluate the mechanical activation of kaolin using response surface methodology (RSM). It analyzed the relationships between rotational speed and grinding time parameters and their effects on amorphous phase content, the reduction in crystalline Kaol, and impurity incorporation into the activated clay, which were evaluated using the Rietveld method.

## 2. Materials and Methods

### 2.1. Materials and Characterization

Commercial kaolin (kaolinitic clay; K) supplied by the Spanish company Minerals i Derivats, S.A. (Tarragona, Spain), was used as the raw material in this study. The composition of K (see Table 1) was determined using X-ray fluorescence (XRF) analysis performed with a Panalytical Philips PW 2400 sequential X-ray spectrophotometer (Malvern Panalytical, Almelo, The Netherlands) equipped with UniQuant^®®^ V5.0 software. The major elements, expressed as their most stable oxides, were silicon and aluminum, with a loss on ignition (LOI) of 12.5 wt.% at 1100 °C. Additionally, the commercial K also contained minor elements such as potassium and iron. 

**Figure 2:**
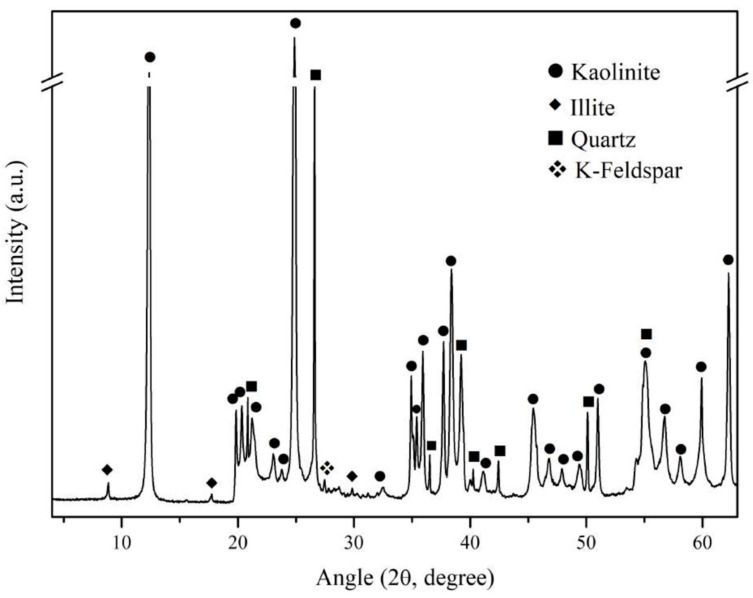
X-ray powder diffractogram of K.

The main crystalline phases of K were identified through X-ray diffraction (XRD) using a Bragg–Brentano PANalytical X’Pert PRO MPD alpha1 powder diffractometer with CuK_α1_ radiation (λ = 1.5406 Å). This featured a Ge (111) primary monochromator, a step size of 0.026°, an anti-scatter slit of 4°, a Soller slit of 0.04 rad, and a measuring time of 100 s per scan, with five repeated scans. The main crystalline phases were detected with the X’Pert HighScore software (version 2.2c). In this sense, kaolinite (PDF# 01-079-1570) and quartz (PDF# 01-085-0695), with weak reflections of microcline (PDF# 01-084-0709), probably with some contribution of other potassium feldspars (such as orthoclase and sanidine), and illite (PDF# 00-026-0911) were identified. Figure 2 illustrates the determined crystalline phases.

A Rietveld refinement was performed using the TOPAS-64 V6 software to quantify the amount of each crystalline phase. The amorphous content (W_amorph_) was estimated with the degree of crystallinity (DOC) function (Equations (1) and (2)), using three peaks to fit the background in TOPAS-64 V6. The content of the crystalline phases was normalized with the degree of crystallinity. Table 2 compiles the results of a semi-quantitative phase quantification analysis.

(1) $$DOC=\frac{Crystalline\;Area}{Cristalline\;Area+Amorphous\;Area}$$

(2) $$W_{amorph}=1-DOC$$

The main crystalline mineral phase identified in K was kaolinite (Kaol), while some reflections of quartz (Q), illite (I), and K-feldspar (K-F) were also observed. The potential transformation of Kaol into metakaolinite through thermal activation, and its subsequent use as an SCM, significantly enhances the properties of the binder. Metakaolin (MK) is a highly reactive pozzolan, making K, with its high Kaol content, an excellent raw material for producing this highly effective SCM. However, the high Kaol content and the difficulty of achieving complete amorphization pose challenges for the mechanical activation of this raw material, highlighting its suitability for this study.

Secondary and backscattered electron images were obtained using a scanning electron microscope (JEOL JSM-7100F, Tokyo, Japan) to analyze the microstructure and morphology of the samples. Additionally, energy-dispersive X-ray spectroscopy (EDS) was conducted at an accelerating voltage of 20 kV and a working distance of 10 mm to precisely analyze the elemental composition of the selected spot area.

Dynamic light scattering (DLS) was employed to evaluate the particle size distribution. The measurements were performed in a laser diffraction particle-sizing analyzer (LS 13 320 from Beckman Coulter) dispersing the clay in ethanol. The specific surface area (SSA) was evaluated with nitrogen as an inert gas (77.3 K) using the Brunauer–Emmett–Teller (BET) method in a TriStar 3000 V6.04.

Furthermore, thermogravimetric analysis (TGA) was conducted using an SDT Q600 device (TA Instruments, New Castle, DE, USA) with a heating rate of 10 °C·min^−1^ up to 1200 °C in a synthetic air atmosphere (50 mL·min^−1^) to investigate the thermal dehydroxylation step of Kaol.

The pozzolanic activity of the activated clay was evaluated using a modified Chapelle test, designed to measure the reactivity of aluminosilicate sources with portlandite [32]. In this procedure, 1 g of activated clay and 2 g of powdered CaO were added to 250 mL of distilled water. The mixture was then vigorously stirred for 16 h at 90 °C using refrigerant equipment for temperature control [33]. A control experiment without the pozzolanic material was conducted under identical conditions. After the reaction system was allowed to cool for 1 h, the solution was combined with 250 mL of 0.7 M sucrose and mixed for 15 min. Subsequently, approximately 150 mL of the reaction mixture was filtered, and a 25 mL aliquot was titrated with 0.1 M HCl in the presence of 3 drops of phenolphthalein. To quantify the fixed portlandite (Ca(OH)_2_), Equation (3) was applied.

(3) $$PA=2\cdot \frac{v_{1}-v_{2}}{v_{1}}\cdot \frac{74}{56}\cdot 1000$$

where PA (pozzolanic activity) refers to the milligrams of fixed Ca(OH)_2_ per gram of activated clay, *v*_1_ is the volume required to titrate 25 mL of the control solution (blank test), and *v*_2_ is the volume required to titrate 25 mL of the solution in which the pozzolanic reaction occurred. For each sample, the test was performed in duplicate, and each solution was titrated per triplicate to ensure repeatability. 

In addition, the R^3^ test was performed to ensure the accuracy of the reactivity measurements. The R^3^ test is based on measuring the heat released during the hydration of a cement-like paste [34]. This test provides a more accurate simulation of cement reactions compared to the modified Chapelle test [35]. The preparation of the R^3^ design mixture was conducted according to ASTM C1897-20 [36]. The activated samples were combined with Ca(OH)_2_ at a 1:3 ratio and CaCO_3_ at a 2:1 ratio. These were then mixed with a solution of K_2_SO_4_ and KOH to form a paste, maintaining a solution-to-solid ratio of 1.2. The solution was made by dissolving 4 g of K_2_SO_4_ and 20 g of KOH in 1 L of distilled water. To make the paste, the solid mixture and solution were stirred together for 2 min at 900 rpm using a high-shear mixer. Then, 15 g of the resulting paste was transferred into a plastic ampoule. The reaction’s heat was monitored over 7 days using a TAM Air isothermal calorimeter (TA Instruments). The calorimeter was set to 40 °C, with a plastic ampoule containing 9.4 g of water used as a reference to prevent any external temperature fluctuations.

### 2.2. Mechanical Activation

The mechanical activations were performed following the methodology of previous studies [27,28]. A planetary ball mill PM 400 (RETSCH, Haan, Germany) equipped with 500-mL zirconia jars and 10-mm zirconia balls was used. The use of 500-mL zirconia jars, instead of the 125-mL jars used in previous research [29], aimed to obtain a larger sample per milling process and to evaluate the effect of jar size on the properties of mechanically activated clay. The jar occupancy was fixed to 20% of the volume with the raw kaolin and balls, and the balls-to-sample mass ratio was also fixed to 20. The formulations were conducted following the experimental design.

### 2.3. Design of Experiments (DoE)

The experimental design methodology, known as the design of experiments (DoE), is an effective approach for organizing experimental work and efficiently evaluating the effects of experimental parameters (variables) on the outcomes (responses) of a process or system [35]. Through an experimental design, information on an experimental domain, defined by the experiments conducted, can be obtained. The outcome of a DoE is a predictive model that enables the determination of the significance of selected variables and their interactions [37].

**Figure 3:**
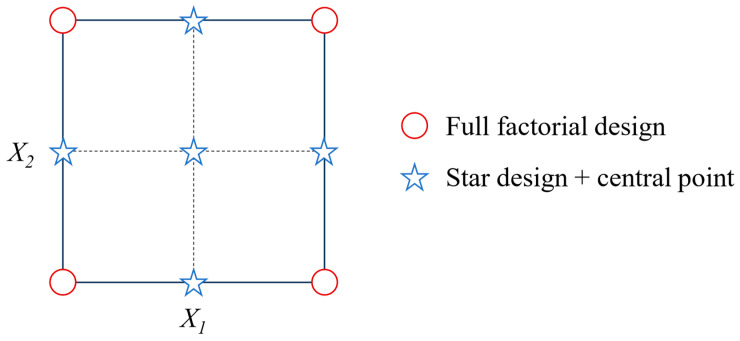
Face-centered central composite design with two variables.

In order to evaluate the parameters influencing the mechanical activation of K at the lab scale, an experimental design was conducted to assess the effects of milling time and rotation speed on the clay. The experimental domain was defined using a central composite design (CCD) with two independent variables (X_1_ and X_2_). This experimental design comprises a full factorial design (2^2^) and a star design (2 × 2) with a central point. A face-centered central composite design (CCF) was chosen, and the star was defined inside the experimental domain, with the points in the faces from the full factorial design. A visual representation of the model used is depicted in Figure 3.

The two variables selected to perform the DoE were rotation speed (X_1_) and milling time (X_2_). Following the CCF design, three levels for each variable were selected: (−1, 0, and +1). The model can be defined using the equation with quadratic terms as follows in Equation (4).

*Y* = *b*_0_ + *b*_1_*X*_1_ + *b*_2_*X*_2_ + *b*_12_*X*_1_*X*_2_ + *b*_11_*X*_1_^2^ + *b*_22_*X*_2_^2^ (4)

where the following applies: *Y* is the response; *b*_0_ is the interception coefficient; *b*_1_, and *b*_2_ are the linear terms; *b*_12_, *b*_11_, and *b*_22_ are the quadratic terms; and *X*_1_ is the rotation speed, *X*_2_ is the milling time, *X*_1_*X*_2_ is the interaction between them, *X*_1_^2^ is the quadratic term of rotation speed, and *X*_2_^2^ is the quadratic term for milling time.

For the rotation speed, the selected levels were 250, 300, and 350 rpm, while for the milling time, the levels were 60, 90, and 120 min. The experiment order was randomized to ensure representative results, with two replicates of each experimental point performed, resulting in a total of 18 experiments. The responses selected to assess the efficiency of the mechanical activation were the contents of the amorphous phase (Y_1_), ZrO_2_ impurity (Y_2_) from zirconia jars and/or milling balls, and crystalline Kaol (Y_3_), all evaluated using the Rietveld method in XRD with the TOPAS software (as described in Section 2.1). Since the main objective of this study was to obtain reactive, mechanically activated K, the goals for these responses were as follows:

i. Maximize the amorphous content (Y_1_), as it is significantly more reactive than crystalline phases. While the amorphous content is mainly from Kaol transformation, the other crystalline phases in K (illite, microcline, and quartz) may also contribute to the total amorphous content through partial or complete amorphization.
ii. Minimize the impurity of ZrO_2_ (Y_2_) to avoid impurities in the material and prevent the deterioration of the milling media.
iii. Achieve a minimum (≈0%; target = 0) of crystalline Kaol (Y_3_) to ensure complete amorphization of the initial Kaol.

The experimental design was conducted using the Design-Expert^®^ 11 software. Table 3 shows a summary of independent variables, ranges, and levels. According to the variables defined in Table 3, the nomenclature used throughout this work for mechanically activated samples is KX_1_-X_2_, where X_1_ and X_2_ refer to the rotation speed (in rpm) and the milling time (in min) for the mechanical activation process, respectively. 

## 3. Results

The resulting experimental matrix based on the CCF experimental design, specified per experimental set, is compiled in Table 4.

### DoE Results

The mathematical models for the three responses were defined using a second-order polynomial regression. The predicted R^2^ values for the responses were as follows: 0.89 for amorphous content (Y_1_), 0.85 for ZrO_2_ impurity percentage (Y_2_), and 0.90 for Kaol content (Y_3_), indicating satisfactory model performance. Table 5 summarizes the significance of the coefficients, and the equations for predicting each response are provided in Equations (5)–(7). Although the *b*_22_ coefficient was not significant for any of the three responses, it was included in the model calculations.

*Y*_1_ = 91.45 − 6.44 *X*_1_ − 3.12 *X*_2_ − 5.12 *X*_1_*X*_2_ − 6.90 *X*_1_^2^ + 0.008 *X*_2_^2^ (5)

*Y*_2_ = 0.73 + 5.70 *X*_1_ + 3.11 *X*_2_ + 4.31 *X*_1_*X*_2_ + 4.97 *X*_1_^2^ + 0.003 *X*_2_^2^ (6)

*Y*_3_ = − 0.006 − 0.66 *X*_1_ − 0.23 *X*_2_ + 0.35 *X*_1_*X*_2_ + 0.66 *X*_1_^2^ + 0.009 *X*_2_^2^ (7)

**Figure 4:**
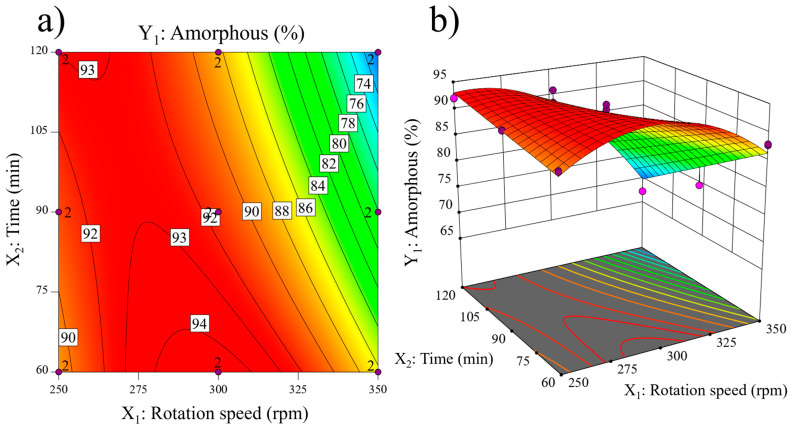
Amorphous content (Y_1_) response: (**a**) contour plot and (**b**) 3D surface.

In Figure 4a, the contour plot illustrates the percentage of amorphous content with contour lines. At constant low milling times, the amorphous content initially increases with increasing rotation speed up to approximately 300 rpm. However, the amorphous content begins to decline at higher rotation speeds. Conversely, at constant high milling times, the amorphous content decreases starting from very low rotation speeds. When the rotation speed is kept constant at low rpm, the amorphous content shows minimal variation, increasing from around 90% to 93%. On the other hand, at a constant rotation speed of 300 rpm, there is a slight decrease in amorphous content with increasing milling time, and at higher speeds, the decrease becomes more pronounced.

Figure 4b presents the 3D response surface for the amorphous content. The purple dots represent the experimental responses of the two replicates, while the surface illustrates the predicted response from the mathematical model. This surface exhibits a parabolic shape, with the amorphous content initially increasing with rotation speed and time and then decreasing after reaching a maximum of around 275–300 rpm and 60 min. The amorphous phase content decreases to its minimum at the corner with 350 rpm and 120 min, indicating the most intense mechanical treatment.

**Figure 5:**
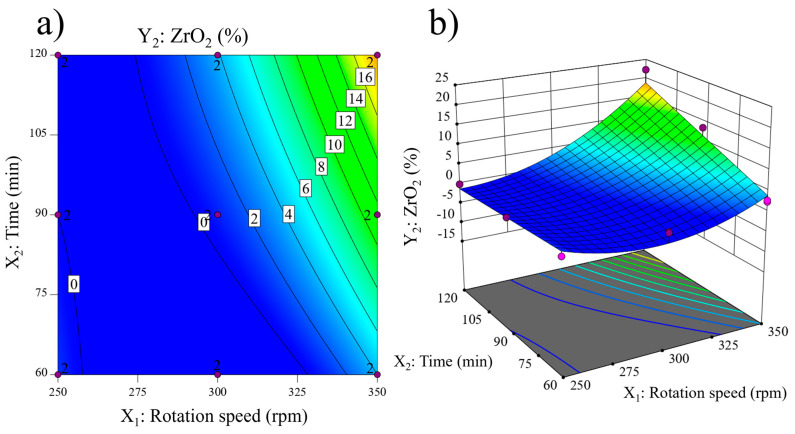
ZrO_2_ impurity content (Y_2_) response: (**a**) contour plot and (**b**) 3D surface.

Figure 5a presents the contour plot for ZrO_2_ impurity. No impurities are detected at low milling times and rotation speeds. The impurity percentage remains minimal across all milling times at a constant low rotation speed. This trend changes at medium rotation speeds (around 300 rpm), where the ZrO_2_ impurity is slightly above 0% at milling times of around 80–90 min and increases with longer milling times. At higher rotation speeds, impurity is detected even at the shortest milling time of the experimental domain (60 min), and the amount of ZrO_2_ significantly increases with milling time. In contrast, with a constant milling time, ZrO_2_ is detected even at the lowest milling time at high rotation speeds (higher than 325 rpm). The impurity content is detected at around 275 rpm at high milling times and significantly increases when rotation speed increases. This behavior is clearly illustrated by the 3D response surface in Figure 5b. The surface is nearly flat at low rotation speeds and milling times, corresponding to 0% ZrO_2_ impurity. The impurity content increases to a maximum at 350 rpm and 120 min, corresponding to the minimum amorphous content. The increase in impurity content with rotation speed is much higher at high milling times than at low milling times. In contrast, the increase in ZrO_2_ content with time is not detected at low rotation speeds, resulting in a flat surface, whereas it is significantly high at high rotation speeds.

**Figure 6:**
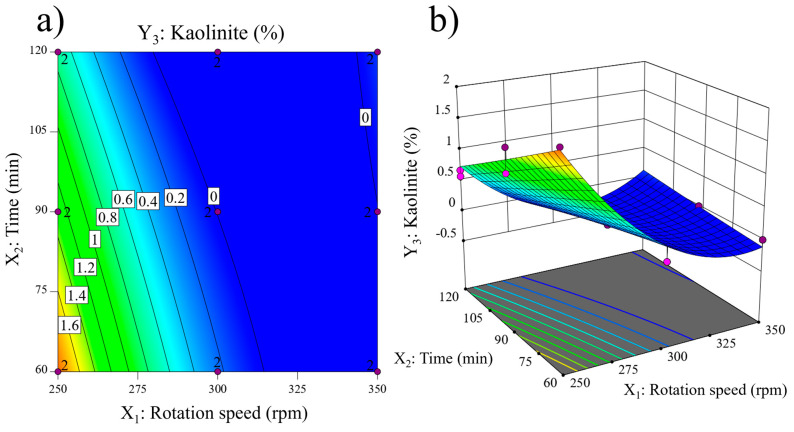
Kaolinite content (Y_3_) response: (**a**) contour plot and (**b**) 3D surface.

The variations in Kaol content are illustrated in the contour plot (Figure 6a). The Kaol content shows an opposite trend to that of ZrO_2_ content: at a constant low rotation speed (250 rpm), Kaol is detected at all milling times. Remarkably, the Kaol content significantly decreases with increasing milling time at low rotation speeds. In contrast, at rotation speeds above 315 rpm, the Kaol content is approximately 0% at all milling times. The 3D surface (Figure 6b) depicts a maximum at 250 rpm and 60 min, corresponding to the least intense mechanical treatment. The curved surface reaches a minimum at around 300 rpm and 90 min and is essentially flat at higher rotation speeds and milling times.

## 4. Discussion

The Kaol structure comprises tetrahedral (T) sheets of SiO_4_ and octahedral (O) sheets of Al(O,OH)_6_. The T- and O-sheets are covalently bonded through apical oxygen atoms, whereas the layers themselves are held together by significantly weaker interlayer forces [38]. Kaol’s TO structure gives rise to two distinct types of hydroxyl groups (OH): inner hydroxyls, situated between the T- and O-sheets within the same layer, and inner-surface hydroxyls, located on the surface of the O-sheet (see Figure 1). These hydroxyl groups are weakly bonded to the adjacent layer’s T-sheet via hydrogen bonds [39].

Mechanical activation induces structural alterations in clay through friction, collision, shear, and other mechanical actions, resulting in products with distinct properties from those of the initial material [40]. Clay mechanical activation consists of two stages, resulting in a progressive loss of crystallinity. During the initial stage, characterized by lower energies or shorter milling times, layered clay minerals undergo delamination and subsequent restacking through the disruption of weak interlayer bonds while retaining the integrity of the structure within each layer [41,42]. In the second stage, the structure within the layers undergoes disruption as covalent bonds fracture, ultimately resulting in amorphization [43,44]. Partially amorphized clay minerals are frequently observed following mechanical activation, indicating the incomplete destruction of their crystalline structure [45]. The structural amorphization of Kaol is attributed to the release of hydroxyl groups from the octahedrally coordinated aluminum layers during mechanical activation [46].

**Figure 7:**
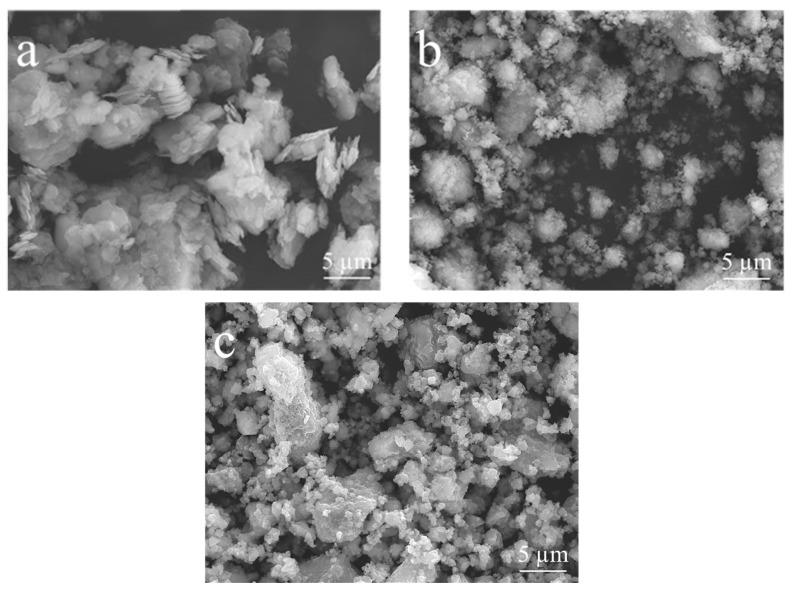
Secondary electron micrographs obtained via SEM of (**a**) K, (**b**) K250-60, and (**c**) K350-120.

The morphological changes resulting from mechanical activation are evident in the secondary electron micrographs obtained via SEM (Figure 7). Initially, K particles exhibited a hexagonal platelet-like morphology (Figure 7a), but this morphology changed with mechanical activation, resulting in distinct rounded equiaxial particles. Furthermore, mechanical activation significantly altered the particle size (Figure 7b,c).

**Figure 8:**
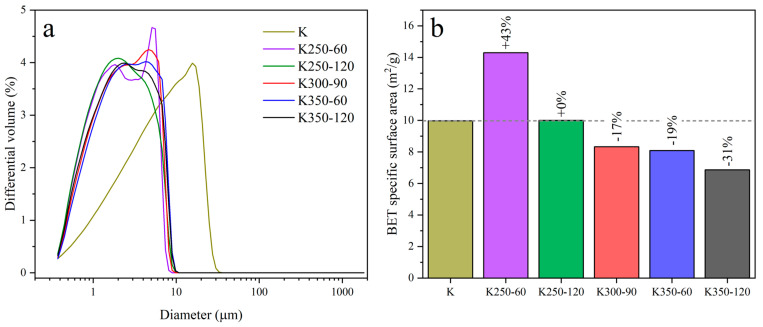
(**a**) Particle size distribution and (**b**) specific surface area of K and representative DoE samples.

K and representative DoE samples were evaluated through DLS to confirm the reduction in particle size (Figure 8a). The selected DoE samples were the four samples located at the vertices of the square in the CCF design (see Figure 3), namely K250-60, K250-120, K350-60, and K350-120, as well as the central point of the experimental domain K300-90. Mechanical activation caused a significant reduction in the particle size of all DoE samples. The particle size distribution of mechanically activated samples displayed a larger volume of particles in the 1–10 µm region than K, with a bimodal distribution. The particles of K250-60 were less affected by milling due to the lower milling time and rotation speed. However, while mechanical activation reduces the particle size, a prolonged milling time or high rotation speed causes the formation of new chemical bonds between particles, leading to agglomeration [28,29,47]. This phenomenon was observed in the SSA results (Figure 8b). With less energetic milling treatments (K250-60), the SSA significantly increased (41% compared to K) due to the particle size reduction and the creation of defects in the activated clay particles. However, increasing the energy of the treatment through the milling time or rotation speed caused a reduction in the SSA. The new chemical bonds and the changes in the pore structure caused by mechanical energy lowered the SSA to up to −31% in K350-120 compared to K.

**Figure 9:**
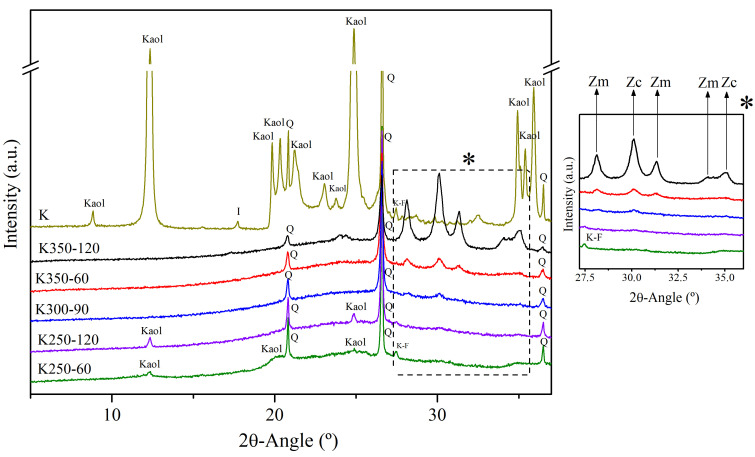
X-ray diffractograms of representative DoE samples: kaolinite (Kaol), illite (I), K-feldspar (K-F), quartz (Q), cubic zirconia (Zc), and monoclinic zirconia (Zm). *: zoomed region 27.5–35.0 2θ°.

The diffractograms of K and representative DoE samples are presented in Figure 9. The amorphous content in mechanically activated clays, observed as a halo in the diffractograms centered around 20–30° 2θ, is a valuable indicator of clay reactivity, regardless of the intended application of the activated K. Consequently, the increased amorphous nature of the clay with intensified mechanical treatment suggests higher reactivity. 

The DoE results corroborate the trends previously identified by the authors [28,29], showing that an increase in milling energy leads to a reduction in Kaol content and a significant increase in the formation of the amorphous phase. Even with less energetic mechanical treatments (K250-60), Kaol was significantly amorphized, with no reflections of illite (I) detected. The K-feldspar (K-F) reflection was only detected at very low speeds and milling times (K250-60), while it disappeared as either of the experimental variables increased. Moreover, a reduction in the intensity of quartz (Q) reflections was observed, becoming more pronounced with increasing milling energy. At high milling speeds and durations (K350-120), Kaol reflections disappeared completely, and a significant reduction in Q reflections was detected. 

However, the DoE results revealed a maximum of amorphous content, beyond which further increases in grinding energy led to a decrease in the amorphous phase content. This reduction is not attributed to an increase in the crystallinity of the clay mineral phases but, rather, to the inclusion of ZrO_2_ impurities from the grinding media and jar, comprising both cubic zirconia (Zc) and monoclinic zirconia (Zm). The ZrO_2_ content reached 22% in K350-120, whereas in the central point of the experimental domain (K300-90), the ZrO_2_ content was only 0.6%. Remarkably, the Kaol content remained minimal across all experiments, peaking at about 2% at the lowest grinding time and rotation speed (K250-60), indicating that all mechanically activated clays should exhibit high reactivity.

**Figure 10:**
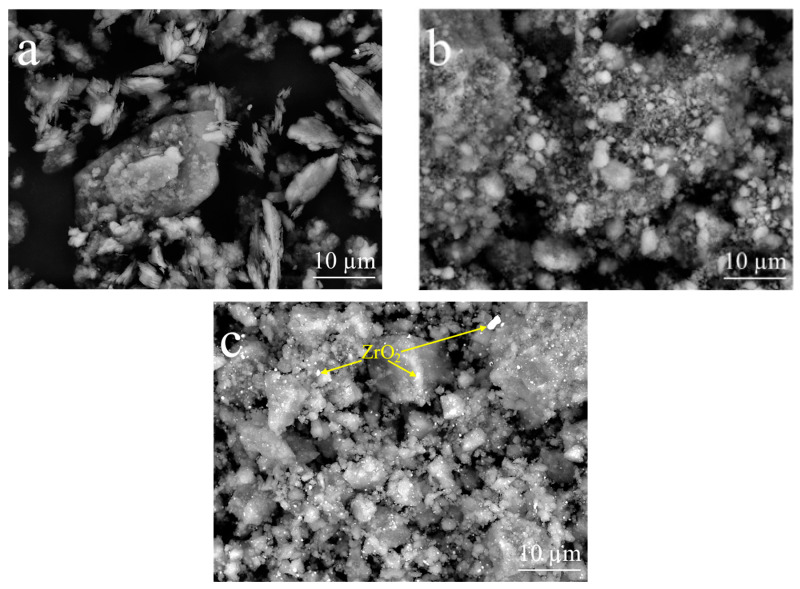
Backscattered electron (BSE) micrographs obtained via SEM: (**a**) K, (**b**) 250-60, and (**c**) 350-120.

The presence of ZrO_2_ in kaolinitic clay samples subjected to prolonged activation times and higher energy levels (e.g., K350-120) is evident in the backscattered electron (BSE) micrographs obtained via SEM (Figure 10). The degree of backscattering significantly depends on the atomic number of the elements present in the sample. Consequently, BSE micrographs provide compositional contrast, with regions of higher atomic numbers scattering more electrons and appearing brighter in the images. In this context, zirconium, having a higher atomic number than aluminum and silicon, appears as brighter areas. Neither the initial kaolin (Figure 10a) nor the mechanically activated clay with relatively low grinding times and energy (e.g., K250-60; Figure 10b) exhibited such bright areas, unlike the K350-120 sample (Figure 10c), which featured elements with high atomic numbers. The zirconia particles were detected to be evenly distributed in K350-120 and had a very small size. 

**Figure 11:**
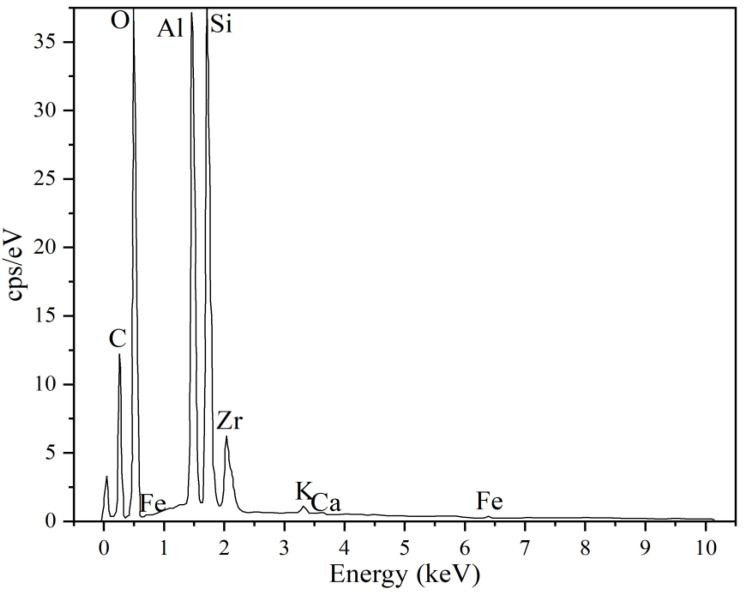
Energy-dispersive X-ray spectroscopy (EDS) analysis of K350-120 sample.

An EDS analysis of the brighter areas observed in BSE micrographs confirmed the presence of zirconium in the high-energy activated clay sample (Figure 11), corroborating the findings previously obtained via XRD (Figure 9). Although the EDS analysis performed was qualitative, the intensity of the EDS peaks suggests a correlation with elemental concentration. In this case, the significant zirconium signal indicates a substantial concentration of zirconium, consistent with the results from the Rietveld analysis of the XRD diffractograms.

**Figure 12:**
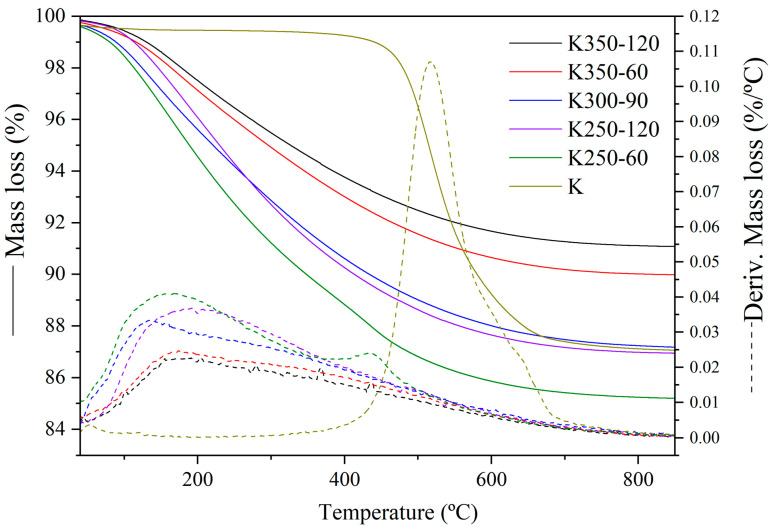
TGA (solid line) and DTG (dashed line) of representative DoE samples.

The modifications of the hydroxyls induced through mechanical activation in the Kaol structure were also supported by TGA and derivative thermogravimetry (DTG). Figure 12 depicts the TGA (solid line) and DTG (dashed line) curves for the five representative samples of mechanical activation experiments. For comparison, the TGA and DTG curves of the raw material (K) are also included. The initial Kaol from K undergoes a dehydroxylation step (400–700 °C), evidenced by the corresponding peak in the DTG, which reaches a maximum at approximately 520 °C. During this thermal process, hydroxyl groups are removed from the structure and released as water vapor. In this case, a shoulder in this peak may also be observed, possibly due to overlapping with illite dehydroxylation, occurring typically at temperatures between 550 °C and 780 °C [44]. However, during the mechanical activation of K, hydroxyl groups from Kaol are not removed but are separated from the structure [46]. With the lower levels of grinding (K250-60), the TGA curve showed a gradual mass loss covering a temperature range of approximately 50 to 700 °C, unlike the distinct step observed in K. However, a minimal dehydroxylation step was also noted in the DTG, accompanied by a reduction in the temperature, indicating that nearly all the Kaol was transformed into amorphous material. This observation is consistent with the corresponding diffractogram of the sample, where kaolinite is still detected in the less activated samples (see Figure 6 and Figure 9). 

The mass loss attributed to the Kaol dehydroxylation process disappeared as the rotation speed and grinding time increased. Instead, a new peak emerged with increasing intensity during mechanical activation, reaching its maximum between 100 °C and 200 °C. This peak corresponds to the loss of non-bonded and weakly bonded OH groups and newly formed H_2_O molecules, which can be either adsorbed or weakly bonded [48]. Some mechanically activated samples exhibited higher total mass loss than K, mainly due to the adsorption of atmospheric water and its reaction with released OH groups, which are released at low temperatures [27]. However, samples subjected to high mechanical energies, such as K350-60 and K350-120, showed a lower total mass loss than K. This difference is attributed to various ZrO_2_ percentages in the samples that dilute the mass loss associated with H_2_O and OH elimination.

**Figure 13:**
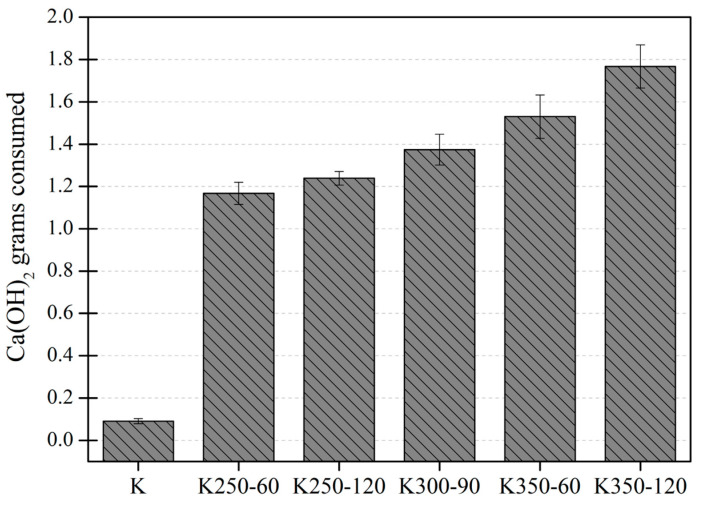
Modified Chapelle test for representative DoE samples and raw material.

The pozzolanic activity of the mechanically activated K samples was assessed using the modified Chapelle test. Figure 13 displays the fixed amount of Ca(OH)_2_ per gram of clay. The pozzolanic activity is mainly attributed to reactive silicon (and aluminum) units from the pozzolanic materials, which react with Ca(OH)_2_ to form calcium silicate hydrates (C-S-H) and/or calcium aluminate silicate hydrates (C-A-S-H) phases. 

There is a significant difference between all mechanically activated samples and the raw clay, emphasizing the effectiveness of the activation process. Furthermore, an increasing trend in the reactivity of the activated samples was detected with an increasing rotation speed or milling time. Specifically, considering that, at 300 rpm and 90 min of milling, the crystalline phases of Kaol and illite disappeared (see Figure 7), it is likely that higher speeds or longer milling times may result in a partial amorphization of quartz. 

The samples obtained at high grinding speeds (350 rpm) exhibit the highest reactivity with portlandite despite a decrease in the total content of amorphous phases due to increased crystalline ZrO_2_ from the grinding balls and jars (see Figure 4 and Figure 5). This increased reactivity should be due to the partial amorphization of quartz and substantial reduction in its particle size, increasing its pozzolanic activity, as detailed by Yao et al. [49]. The presence of a higher amount of reactive silica in the DoE samples with the most energetic mechanical treatment could enhance the formation of C-S-H, which is the main reaction product of activated clay with portlandite, hence consuming more portlandite in the modified Chapelle test.

**Figure 14:**
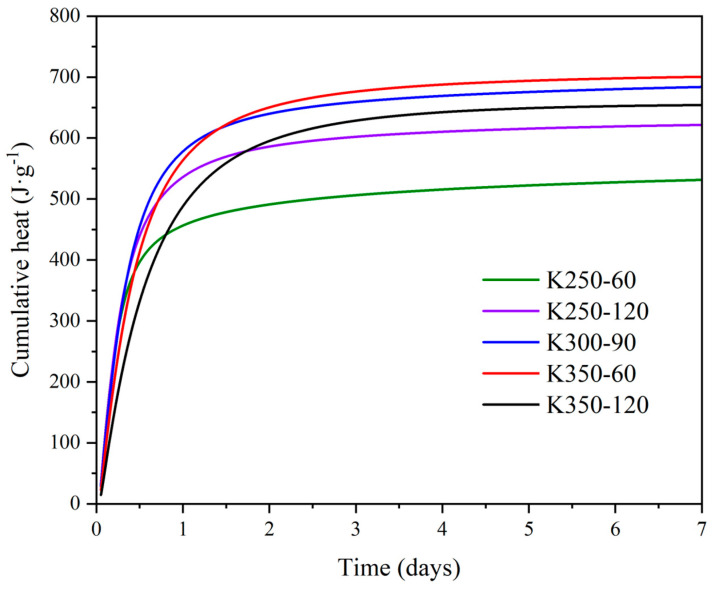
R^3^ test heat release for representative DoE samples.

The R^3^ test was implemented to corroborate the pozzolanic activity of the mechanically activated K samples. This test measures the heat the SCM reaction system releases for 7 days. Figure 14 compiles the cumulative heat of the R^3^ test of representative DoE samples, measured in J⋅g^−1^ of SCM. K250-60 presented the lowest cumulative heat at around 500 J⋅g^−1^ due to the lower degree of amorphization. K250-120, K300-90, K350-60, and K350-120 fell in the same range of heat released, between 600 J⋅g^−1^ and 700 J⋅g^−1^. K300-90 and K350-60 exhibited higher pozzolanic activity than K350-120, likely caused by the presence of ZrO_2_ in K350-120, which effectively reduced its reactivity by reducing the content of amorphous phase (see Figure 4 and Figure 5). Furthermore, the reduction in the SSA could also decrease the reactivity of the activated clays (see Figure 8). 

The decreased pozzolanic activity in K350-120 was not observed in the modified Chapelle test results, probably because the system contained only Ca(OH)_2_, favoring the reaction of silica, rather than alumina, to form C-S-H. Therefore, a partial amorphization of quartz could increase the portlandite consumption despite the inclusion of zirconia in the sample. In contrast, the R^3^ test contains sulfate and carbonate ions, which react with alumina to form ettringite (or monosulfate) and carboaluminates. Therefore, the potentially higher reactive silica content would not lead to a much higher reactivity of K350-120, while the presence of ZrO_2_ and low SSA would decrease the pozzolanic activity.

The treatments entailing higher energy consumption and significant wear and tear on the grinding equipment (and increased impurities in the sample) could pose a drawback at the industrial scale. Meanwhile, samples such as K250-120 or K350-60 exhibited high reactivity using only 250 rpm or 60 min, thereby reducing energy consumption and preserving the grinding equipment while achieving similar performances.

## 5. Conclusions

The design of experiments (DoE) results demonstrate that mechanical activation at the laboratory scale is highly effective for preparing clays to use as supplementary cementitious materials (SCMs) for the formulation of low-carbon cements. The study reveals that it is feasible to adjust grinding parameters, such as the rotation speed and milling time, to achieve a high degree of clay activation.

These adjustments enhance the content of amorphous phases, particularly from kaolinite (Kaol) and, to a lesser extent, illite, while substantially reducing the particle size. Notably, it is unnecessary to apply the most intensive grinding parameters in order to achieve a substantial amorphization of the clay’s mineral phases. Intermediate rotation speeds and grinding times are sufficient to yield a high content of amorphous material and suitably sized particles, ensuring the activated clay’s reactivity. The pozzolanic activity of the mechanically activated clay increases with higher rotation speeds and longer grinding times in a planetary ball mill. However, highly energetic grinding introduces a significant increase in impurities, such as ZrO_2_, due to the wear of the grinding balls and jar and causes a substantial reduction in the specific surface area. This may lead to lower clay performance as SCM.

Consequently, overly aggressive grinding processes may be detrimental to clay activation. An optimal balance must be reached to produce highly reactive clay while minimizing impurities, energy consumption, and wear on milling equipment. This balance ensures effective mechanical activation without compromising the integrity of grinding equipment or incurring excessive costs. In this context, the DoE methodology has proven helpful in selecting suitable working parameters, regardless of the mill or milling conditions used. Therefore, the methodology proposed in this study could be useful for scaling the mechanical activation technology up to the industrial level. 

## Figures and Tables

**Table 1:** Chemical characterization of the raw kaolin.

Compound	SiO_2_	Al_2_O_3_	K_2_O	Fe_2_O_3_	CaO	TiO_2_	Na_2_O	MgO	P_2_O_5_	LOI ^a^
wt.%	49.85	36.31	0.69	0.47	0.16	0.15	0.13	0.11	0.08	12.50

^a^ LOI: loss on ignition at 1100 °C.

**Table 2:** Crystalline phases and amorphous content estimation of K.

Mineral Phases	(wt.%)
Kaolinite	82.0
Quartz	5.7
Illite	5.3
K-feldspar	1.6
Amorphous content	5.4

**Table 3:** Experimental range and levels of independent process variables.

Independent Variables	Symbol	Range and Levels		
		−1	0	+1
Rotation speed (rpm)	X_1_	250	300	350
Milling time (min)	X_2_	60	90	120

**Table 4:** Experimental matrix, independent variables, and responses. X_1_: rotation speed; X_2_: milling time; Y_1_: amorphous content; Y_2_: ZrO_2_ impurity; and Y_3_: crystalline Kaol.

Experiment	X_1_ (rpm)	X_2_ (Min)	Y_1_ (%)	Y_2_ (%)	Y_3_ (%)
1	250 (−1)	60 (−1)	89.92	-	1.94
2	250 (−1)	60 (−1)	89.71	-	2.11
3	350 (+1)	60 (−1)	87.73	2.46	-
4	350 (+1)	60 (−1)	87.94	2.83	-
5	250 (−1)	120 (+1)	91.92	-	0.67
6	250 (−1)	120 (+1)	92.05	-	0.57
7	350 (+1)	120 (+1)	67.24	22.25	-
8	350 (+1)	120 (+1)	71.81	17.48	-
9	250 (−1)	90 (0)	91.26	-	1.10
10	250 (−1)	90 (0)	91.09	-	1.49
11	350 (+1)	90 (0)	74.54	13.38	-
12	350 (+1)	90 (0)	79.40	10.00	-
13	300 (0)	60 (−1)	91.72	0.31	-
14	300 (0)	60 (−1)	92.82	0.03	-
15	300 (0)	120 (+1)	88.51	2.00	-
16	300 (0)	120 (+1)	90.88	1.17	-
17	300 (0)	90 (0)	91.94	0.57	-
18	300 (0)	90 (0)	92.85	0.30	-

**Table 5:** Coefficients and coefficients’ significance of the experimental face-centered central composite design (CCF).

Coefficient	Y_1_		Y_2_		Y_3_	
	Value	*p*-Value	Value	*p*-Value	Value	*p*-Value
*b*_0_	91.45		0.73		−0.01	
*b*_1_	−6.44	<0.0001	5.70	<0.0001	−0.66	<0.0001
*b*_2_	−3.12	0.0002	3.11	0.0002	−0.23	0.0010
*b*_12_	−5.12	<0.0001	4.31	<0.0001	0.35	0.0002
*b*_11_	−6.90	<0.0001	4.97	0.0005	0.66	<0.0001
*b*_22_	0.01	0.9944 *	0.00	0.9981 *	0.01	0.9240 *

* Not significant coefficients.

## Data Availability

The data supporting this study are available from the corresponding author upon request.
